# TES inhibits colorectal cancer progression through activation of p38

**DOI:** 10.18632/oncotarget.9961

**Published:** 2016-06-13

**Authors:** Huili Li, Kun Huang, Lu Gao, Lixia Wang, Yanfeng Niu, Hongli Liu, Zheng Wang, Lin Wang, Guobin Wang, Jiliang Wang

**Affiliations:** ^1^ Department of Gastrointestinal Surgery, Union Hospital, Tongji Medical College, Huazhong University of Science and Technology, Wuhan, Hubei, China; ^2^ Institution of Cardiology, Union Hospital, Tongji Medical College, Huazhong University of Science and Technology, Wuhan, Hubei, China; ^3^ Department of Cardiology, The First Affiliated Hospital of Zhengzhou University, Zhengzhou, Henan, China; ^4^ Department of Radiology, Union Hospital, Tongji Medical College, Huazhong University of Science and Technology, Wuhan, Hubei, China; ^5^ Cancer Center, Union Hospital, Tongji Medical College, Huazhong University of Science and Technology, Wuhan, Hubei, China; ^6^ Research Center for Tissue Engineering and Regenerative Medicine, Union Hospital, Tongji Medical College, Huazhong University of Science and Technology, Wuhan, Hubei, China

**Keywords:** TESTIN gene, tumor suppressor gene, colorectal cancer, HCT116 cell and DLD-1 cell, p38 MAP kinase

## Abstract

The human TESTIN (TES) gene has been identified as a candidate tumor suppressor based on its location at a common fragile site – a region where loss of heterozygosity has been detected in numerous types of tumors. To investigate its role in colorectal cancer (CRC), we examined TES protein levels in CRC tissue samples and cell lines. We observed that TES was markedly reduced in both CRC tissue and cell lines. Additionally, overexpression of TES significantly inhibited cell proliferation, migration, and invasion, while increasing cell apoptosis in colon cancer cells. By contrast, shRNA-mediated TES knockdown elicited the opposite effects. TES inhibited the progression of CRC by up-regulating pro-apoptotic proteins, down-regulating anti-apoptotic proteins, and simultaneously activating p38 mitogen-activated protein kinase (MAPK) signaling pathways. Collectively, these data indicate that TES functions as a necessary suppressor of CRC progression by activating p38-MAPK signaling pathways. This suggests that TES may have a potential application in CRC diagnosis and targeted gene therapy.

## INTRODUCTION

Colorectal cancer (CRC) is one of the most common malignancies worldwide [1–3], with a rising incidence and high mortality and morbidity rates [4, 5]. CRC is the third leading cause of cancer death, accounting for over 50,000 deaths worldwide in 2014 [2, 3, 5]. Although progress has been made in the molecular characterization of CRC over the last decade [6, 7], the options for CRC therapy are still limited in some senses, and the therapeutic effect is still not satisfactory [8, 9]. Better understanding of the molecules and signal transduction pathways involved would facilitate the development of more effective therapeutic strategies [7, 9], bringing potential improvements for outcomes and quality of life [10].

The human TESTIN gene, also known as TES, has been implicated in cancer due to its loss of expression in prostate cancer [11, 12], its presence at the sites of focal adhesions, and its role in cytoskeletal organization [13–18]. TES was mapped to a common fragile site at chromosome 7q31.1/2, designated as FRA7G. Deletions at 7q31.1/2 have been detected in several types of leukemia as well as tumors of the breast, ovary, prostate, colon, stomach, kidney, head, and neck [11, 12, 19]. Loss of gene expression within this region may play a role in the development and progression of neoplasia. Loss of FRA7G heterozygosity is seen in many human malignancies, and some studies suggest that genetic and epigenetic alterations of one or more tumor suppressor genes in the FRA7G region, potentially including TES, are involved in multiple malignancies [20, 21].

TES is expressed in all normal human tissues [22]. It is localized in the cytoplasm as a component of focal adhesions and cell-cell connections [13–18]. Lack of TES mRNA expression was found in several cancer-derived cell lines, particularly hematopoietic, breast, prostate, and ovarian cancer cell lines as well as in primary tumors. This phenomenon was found to be correlated with the methylation of CpG islands in the TES gene [20, 21]. The functional role of TES in CRC remains unclear [20]. In this study we sought to uncover the connection between TES and CRC first by comparing its expression in CRC tissue samples and adjacent tumor-free samples and in CRC cell lines and then to determine the effect of manipulating TES expression on CRC cell behavior *in vitro* and *in vivo*.

## RESULTS

### Loss of TES expression in CRC tissue samples and cell lines

**Figure 1:**
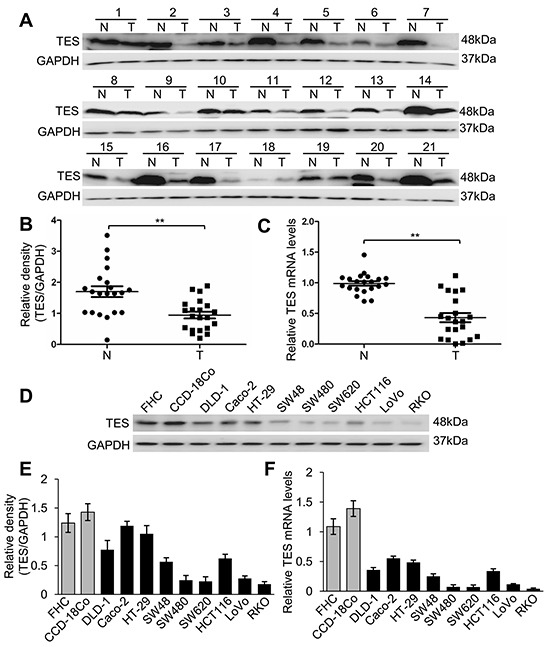
Loss of TES expression in CRC tissues and cell lines **A.** Western blot analysis of TES protein levels in 21 paired CRC tissue and adjacent tumor-free tissue samples. **B.** Densitometric analysis of Western blot of TES in CRC tissue and adjacent tumor-free tissue samples. **C.** Relative expression of TES mRNA evaluated by real-time qPCR in CRC tissue and adjacent tumor-free tissue samples. N represents corresponding adjacent tumor-free tissue; T represents CRC tissue. **D.** Western blot analysis of TES protein levels in two normal human colon cell lines (FHC and CCD-18Co) and nine human CRC cell lines (DLD-1, Caco-2, HT-29, SW48, SW480, SW620, HCT116, LoVo and RKO). **E.** Densitometric analysis of Western blot of TES in normal colon cells and CRC cells. **F.** Relative expression of TES mRNA evaluated by real-time qPCR in normal colon cells and CRC cells. ** *p* < 0.01. Data are plotted as the mean ± SD from five independent experiments. Bars indicate the standard deviation of the mean.

TES expression profiles from 21 pairs of human CRC tissue samples and the corresponding adjacent tumor-free tissue samples (Supplementary Table S1) were examined by Western blot analysis and real-time quantitative PCR (real-time qPCR). TES protein levels (*p* < 0.01; Figure 1A and 1B) and mRNA expression (*p* < 0.01; Figure 1C) were significantly reduced in CRC tissue samples compared with the adjacent tumor-free tissue samples. Additionally, we analyzed the correlation with histological grades or TNM stages in CRC samples and found no correlation between TNM stages and TES protein levels (data not shown). However, a significant adverse correlation between histological grades and TES protein levels was observed (*p* < 0.01; Supplementary Figure S1A).

We then examined TES mRNA expression and protein level in a panel of nine CRC cell lines (DLD-1, Caco-2, HT-29, SW48, SW480, SW620, HCT116, LoVo, and RKO) and two kinds of normal human colon cells (FHC as a normal human colon epithelial cell and CCD-18Co as a normal human colon cell). TES protein (Figure 1D and 1E) and mRNA (Figure 1F) were remarkably reduced in CRC cell lines compared with the two kinds of normal human colon cells. We also analyzed the correlation between Broder's grade and Duke's classification of the original tumors and the TES protein levels in the CRC cell lines [23–25]. Duke's classification of four cell lines could not be determined; based on the remaining five cell lines, no correlation could be found between Duke's classification of the original tumors and the TES protein levels (data not shown). However, a significant adverse correlation between histological grades of the original tumors and TES protein levels was observed (*p* < 0.01; Supplementary Figure S1B).

### TES-suppressed cell proliferation, migration, and invasion of CRC cells *in vitro*

**Figure 2:**
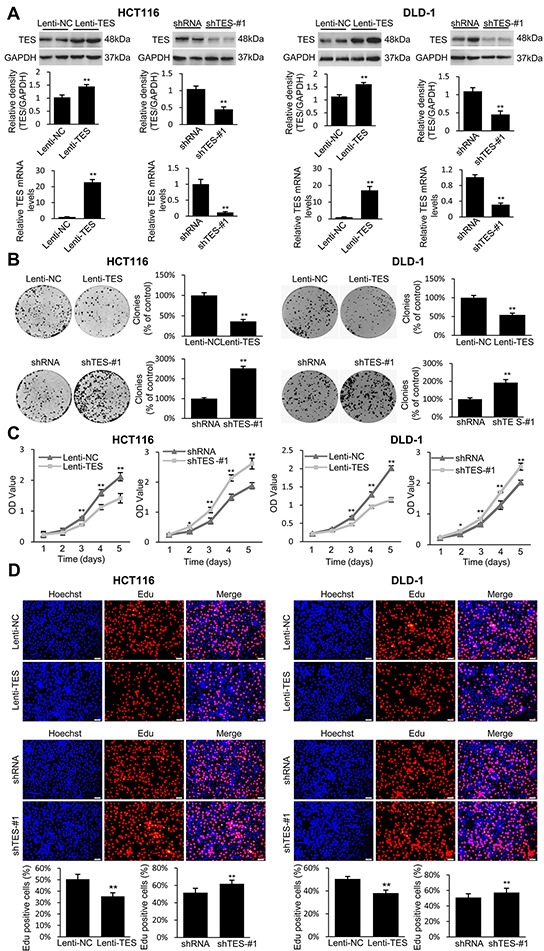
TES suppresses proliferation in CRC cells **A.** Confirmation of the protein levels and mRNA expression of TES in the genetically modified HCT116 and DLD-1 cells by Western blot and real-time qPCR. GAPDH was used as endogenous control. **B.** Representative photographs of cell culture plates following staining with crystal violet for colony formation of the genetically modified HCT116 and DLD-1 cells. Number of colonies was quantified and plotted as percent (%) relative to the control. **C.** Cell growth of the genetically modified HCT116 and DLD-1 cells was determined by MTT assays at each time point. **D.** Representative profiles of EdU cell proliferation assay in the genetically modified HCT116 and DLD-1 cells. Representative photographs were taken at 200× magnification.* *p* < 0.05; ** *p* < 0.01. Data are plotted as the mean ± SD from five independent experiments. Bars indicate the standard deviation of the mean.

**Figure 3:**
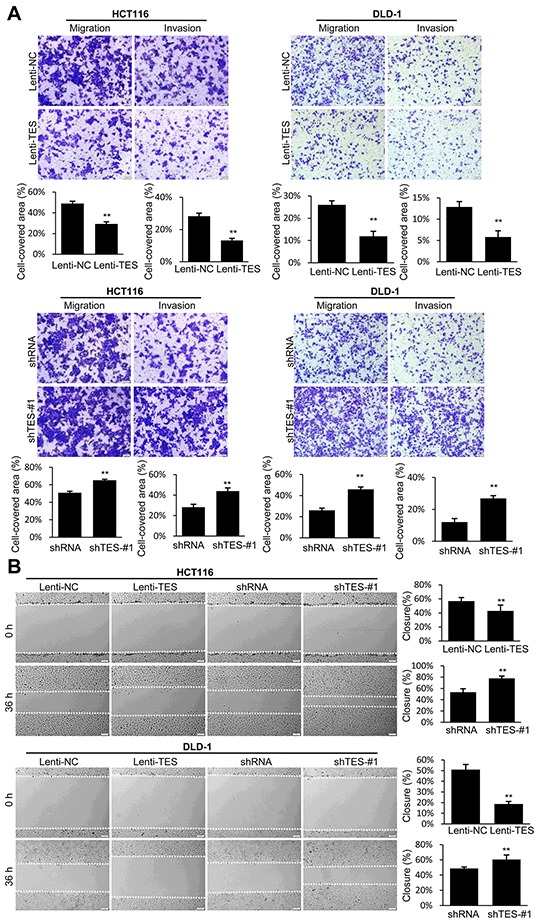
TES suppresses migration and invasion in CRC cells **A.** Cell migration and invasion was assessed after 24 h incubation by Transwell^®^ assay. **B.** Representative images of wound healing assay by scraping culture dishes using a pipet tip and closure after 36 h of culture. Representative photographs were taken at × 100 magnification. ** *p* < 0.01. Data are plotted as the mean ± SD from five independent experiments. Bars indicate the standard deviation of the mean.

**Figure 4:**
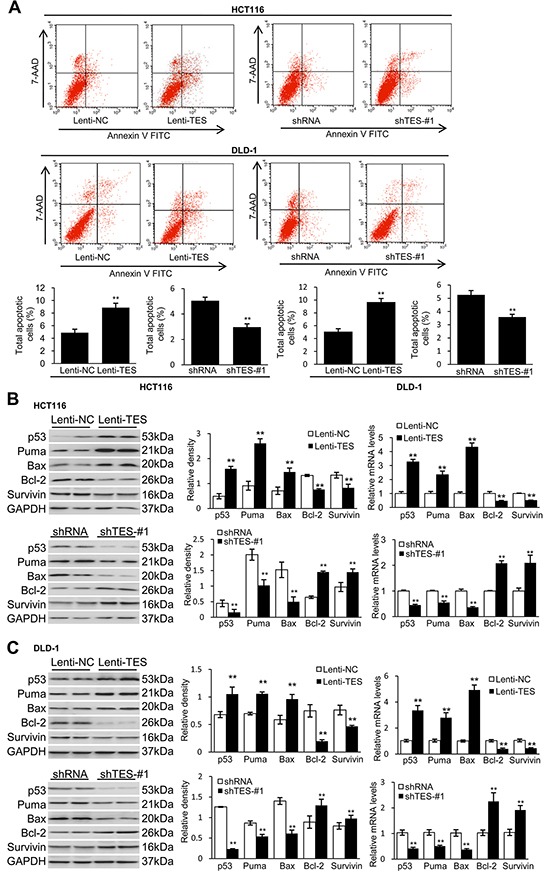
TES promotes apoptosis in CRC cells The genetically modified HCT116 and DLD-1 cells were grown over time respectively. **A.** Cell apoptosis was measured by flow cytometric analyses. Representative biparametric histogram showing cell population in apoptotic (top right and bottom right quadrants), viable (bottom left quadrant) and necrotic (top left quadrant) states. **B.** Protein levels and mRNA expression of p53, Puma, Bax, Bcl-2 and survivin of HCT116 cells. **C.** Protein levels and mRNA expression of p53, Puma, Bax, Bcl-2 and survivin of DLD-1 cells. GAPDH was used as loading control. ** *p* < 0.01. Data are plotted as the mean ± SD from five independent experiments. Bars indicate the standard deviation of the mean.

In order to explore the function of TES in CRC further, we established stable cells that constitutively overexpressed TES using a lentiviral vector and generated TES-knockdown cell line models by stably expressing knockdown constructs (TES-specific shRNAs) in the CRC cell lines HCT116 and DLD-1. The transfection efficiency was confirmed using Western blot and real-time qPCR analysis. As shown in Figure 2A, the HCT116 and DLD-1 cells transfected with the overexpression plasmid (Lenti-TES cell lines) showed significantly increased TES protein and mRNA compared with cells transfected with empty vector (Lenti-NC cell lines). The HCT116 and DLD-1 cells stably expressing the knockdown constructs (shTES cell lines) showed significantly decreased TES protein and mRNA compared with cells transfected with control plasmid (shRNA cell lines). We used two shRNAs (shTES-#1 and shTES-#2) targeting different TES sequences to reduce the possibility that the phenotypes observed were due to off-target effects of the shRNAs. The data for shTES-#1 are shown in Figures 2, 3, and 4, whereas the data for shTES-#2 are only shown in Supplementary Figures due to the lower efficiency of shTES-#2.

After establishing ten stably transfected cell lines (Lenti-NC-HCT116, Lenti-TES-HCT116, shRNA-HCT116, shTES-#1-HCT116, and shTES-#2-HCT116; Lenti-NC-DLD-1, Lenti-TES-DLD-1, shRNA-DLD-1, shTES-#1-DLD-1, and shTES-#2-DLD-1), we first performed a clonogenic assay. As shown in Figure 2B (and Supplementary Figure S2B), overexpression of TES in HCT116 and DLD-1 cells dramatically reduced the colony formation efficiency *in vitro*, whereas the colony formation efficiency was dramatically increased in the TES-knockdown cells compared with control cells (*p* < 0.01).

We next investigated the effect of TES expression on cell growth and proliferation. As shown in Figure 2C (and Supplementary Figure S2C), MTT assay revealed that TES overexpression significantly inhibited cell growth, whereas knockdown of TES significantly enhanced the growth of HCT116 and DLD-1 cells. In order to characterize the effect of TES expression on the proliferation of HCT116 and DLD-1 cells further, an EdU proliferation assay was performed. After culturing for 24h, the proliferation rate of the ten kinds of cancer cells were measured (Figure 2D and Supplementary Figure S2D). These data suggest that TES contributed to the reduced growth or proliferation in CRC cells.

We examined the effect of TES on cell migration and invasion. We observed that the Lenti-TES cells displayed significantly reduced cell migration and invasion compared with Lenti-NC cells 24 h after seeding in the uncoated as well as Matrigel™-coated Transwell^®^ chambers (Figure 3A and Supplementary Figure S2E) and 36 h after scratch was performed (Figure 3B and Supplementary Figure S2F). In contrast, enhanced cell migration and invasion was observed in shTES cells compared with control shRNA cells, as shown in Figure 3 and Supplementary Figure S2E and S2F.

Together, these data suggest that TES plays a significant role in suppressing the proliferation, migration, and invasion of CRC cells *in vitro*.

### TES promotes apoptosis of CRC cells

In addition to the observed suppression effects of TES on proliferation, migration, and invasion in CRC cells, we studied whether TES could affect apoptosis in CRC cells. Flow cytometry showed a significantly increased apoptotic cell fraction (Annexin V+/7-AAD±) in Lenti-TES cells compared with Lenti-NC cells after 48 h culture in medium without fetal bovine serum (FBS; 8.87 ± 0.69% versus 4.88 ± 0.54%, *p* < 0.01; Figure 4A). In contrast, shTES-#1 and shTES-#2 cells exhibited obviously decreased apoptotic cell fractions compared with shRNA cells. (2.97 ± 0.26% versus 5.05 ± 0.27%, *p*<0.01; 3.26 ± 0.26% versus 5.26 ± 0.25%, *p* < 0.01; Figure 4A and Supplementary Figure S3).

To investigate how TES induces CRC cell apoptosis, we studied the expression of apoptosis-related proteins in the modified HCT116 and DLD-1 cell lines. Western blot and real-time qPCR analysis showed increased protein levels and mRNA expression of pro-apoptotic proteins (p53, Puma, and Bax) and decreased protein levels and mRNA expression of anti-apoptotic proteins (Bcl-2 and survivin) in Lenti-TES cells compared with Lenti-NC cells (Figure 4B and 4C). In contrast, the TES-knockdown cells displayed the opposite pattern (Figure 4B and 4C).

### TES suppresses tumor formation and growth of CRC cells *in vivo*

**Figure 5:**
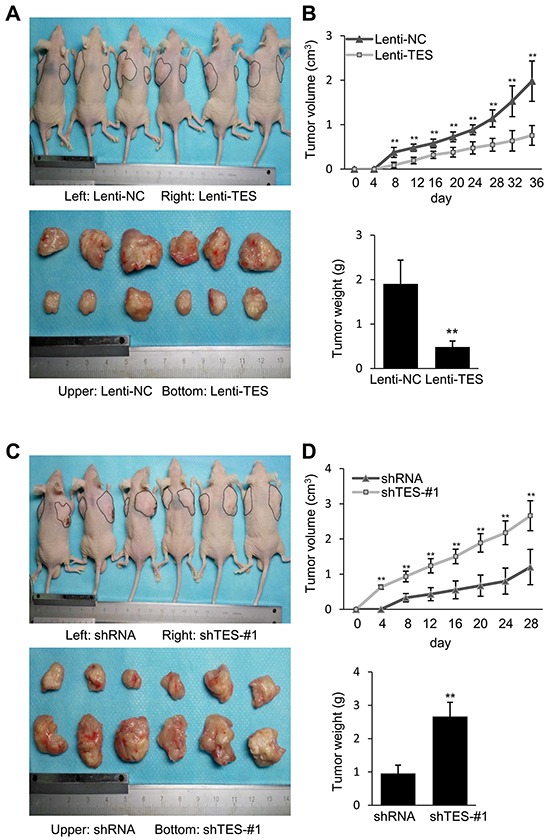
TES suppresses tumor formation and growth of CRC cells *in vivo* **A.** Photographs of tumors in mice and excised 36 days after inoculation of stably transfected Lenti-NC and Lenti-TES HCT116 cells into athymic nude mice. **B.** Growth of tumor volume plotted over time, weight of tumor at the end of 36 days were plotted of six mice per group. **C.** Photographs of tumors in mice and excised 28 days after inoculation of stably transfected shRNA and shTES-#1 HCT116 cells into athymic nude mice. **D.** Growth of tumor volume plotted over time, weight of tumor at the end of 28 days were plotted of six mice per group. ** *p* < 0.01. Data are shown as mean ± SD. Bars indicate the standard deviation of the mean.

To determine the suppressive effect of TES in CRC cells *in vivo*, Lenti-TES-HCT116, Lenti-NC-HCT116, shTES-#1-HCT116, or shRNA-HCT116 cells were subcutaneously injected into nude mice. We observed that TES overexpression remarkably inhibited tumor formation and growth *in vivo* (Figure 5A), whereas TES knockdown significantly facilitated tumor formation and growth *in vivo* (Figure 5C), evaluated by the volume and weight of xenograft tumors (Figure 5B and 5D). These findings suggest that TES functions as a tumor suppressor in CRC cells *in vivo*.

### TES activates p38-MAPK signaling pathways in HCT116 and DLD-1 cells

**Figure 6:**
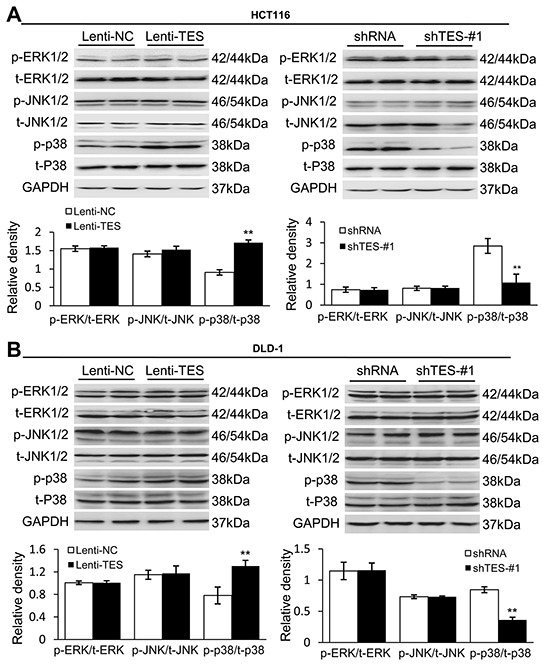
TES functions as a tumor suppressor by activating the p38-MAPK signaling pathway **A.** Representative Western blots showing the phosphorylation and total protein levels of ERK1/2, JNK1/2 and p38 in the genetically modified HCT116 cells. **B.** Representative Western blots showing the phosphorylation and total protein levels of ERK1/2, JNK1/2 and p38 in the genetically modified DLD-1 cells. GAPDH was used as loading control. ** *p* < 0.01. Data are plotted as the mean ± SD from five independent experiments. Bars indicate the standard deviation of the mean.

Finally, we performed Western blot analysis to analyze the signaling pathways involved in TES-mediated inhibition of proliferation, migration, and invasion in HCT116 and DLD-1 cells. We first examined whether TES affected the MAPK signaling pathways, as these have previously been shown to play an important role in CRC [26, 27]. P38 phosphorylation levels were dramatically elevated in Lenti-TES cells compared with Lenti-NC cells, whereas extracellular signal-regulated kinase (ERK) and Jun N-terminal Kinase (JNK) were not significantly affected (Figure 6). Conversely, the phosphorylation of p38 was almost completely impaired in shTES cells compared with shRNA cells (Figure 6). Although the AKT signaling pathway plays a crucial role in the regulation of CRC [28, 29], we did not observe any differences in the AKT signaling pathways of these cells (data not shown). These data suggest that the TES regulation of CRC may be mediated by the p38-MAPK signaling pathway rather than the AKT pathway.

### Inhibition of p38 activity reverses tumor suppression in TES-overexpressing cells

**Figure 7:**
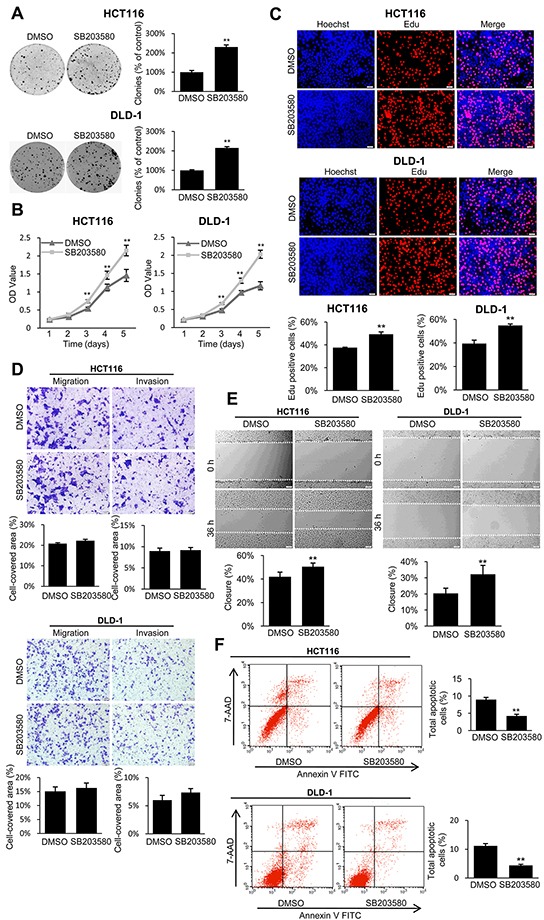
The p38 inhibitor reversed the tumor suppressive effect of TES in CRC cells **A.** Representative photographs of cell culture plates following staining with crystal violet for colony formation of the TES-overexpressing HCT116 and DLD-1 cells in the presence or absence of p38 inhibitor. Number of colonies was quantified. **B.** The cell growth of the TES-overexpressing HCT116 and DLD-1 cells in the presence or absence of p38 inhibitor were determined by MTT assays at each time point. **C.** Representative profiles of EdU cell proliferation assay of the TES-overexpression HCT116 and DLD-1 cells in the presence or absence of p38 inhibitor. Representative photographs were taken at 200× magnification. **D.** Cell migration and invasion was assessed after 24 h incubation in the presence or absence of p38 inhibitor by transwell assays. Representative photographs were taken at 100× magnification. **E.** Representative images of wound healing assay by scraping culture dishes using a pipet tip and closure in the presence or absence of p38 inhibitor after 36 h of culture. Representative photographs were taken at 100× magnification. **F.** Cell apoptosis of TES-overexpressing HCT116 and DLD-1 cells in the presence or absence of p38 inhibitor was measured by flow cytometric analyses. Representative biparametric histogram showing cell population in apoptotic (top right and bottom right quadrants), viable (bottom left quadrant) and necrotic (top left quadrant) states. ** *p* < 0.01. Data are plotted as the mean ± SD from five independent experiments. Bars indicate the standard deviation of the mean.

To confirm whether p38 mediated the tumor suppressive effects of TES in CRC, we chose SB203580, a specific inhibitor of p38 MAP kinase, to treat the TES-overexpressing cells (Lenti-TES-HCT116 and Lenti-TES-DLD-1). As shown in Figure 7 and Supplementary Figure S4, treatment with SB203580 markedly promoted growth or proliferation (Figure 7A, 7B, 7C, and Supplementary Figure S4) and inhibited apoptosis (Figure 7F), but only slightly affected migration or invasion in TES-overexpressing cells (Figure 7D and 7E). These results suggest that p38 activation is critical for the anti-proliferation and pro-apoptosis effect of TES in colon cancer cells, while the migration and invasion suppressive effect may be mediated by the interaction between TES and cytoskeleton proteins.

## DISCUSSION

CRC has one of the highest incidences and mortality rates of all cancers worldwide [1-5, 30]. The formation of CRC is a multistep process that may be induced by the accumulation of genetic or epigenetic alterations [31, 32]. These alterations tend to occur in chromosomal fragile regions, which can provide a strategy to discover cancer susceptibility genes [33, 34]. The human TES gene is located at 7q31.1/2 within the fragile chromosomal region FRA7G, which has been reported to be involved in the loss of heterozygosity in several human malignancies [11, 20, 21, 35–38]. The expression of TES is downregulated in a variety of tumor cell lines, particularly hematopoietic, breast, and ovarian cancer cell lines as well as in primary tumors [20, 21].

In the present study, we initially evaluated the expression of TES in CRC and adjacent tumor-free tissue samples as well as in various CRC cell lines and normal colon cells. Western blot analysis and real-time qPCR demonstrated that CRC specimens and all of the nine kinds of colon cancer cells exhibited significantly reduced TES protein and mRNA compared with the adjacent tumor-free tissue samples and the two kinds of normal colon cells. Furthermore, there is a significant adverse correlation between degrees of malignancy in the cancer samples (or the original tumors of the cancer cell lines) and TES protein levels. These results suggest that loss of TES plays an important role in CRC tumorigenesis and progression.

It has been demonstrated that overexpression of TES suppresses tumor cell growth and induces apoptosis in breast and uterine cancer [19, 20, 22]. Moreover, the deletion of TES is associated with hematopoietic malignancies and epithelial tumors. Taken together, these data implicate TES as a putative tumor suppressor gene [19]. To confirm TES tumor suppressor function in CRC, we chose HCT116 and DLD-1 cells for their moderate TES protein levels, and then established colon cancer cell lines that stably overexpress TES or shRNA-targeting TES. Our studies demonstrate that TES suppresses the proliferation, migration, and invasion of CRC cells *in vitro* and inhibits the tumor growth of CRC cells *in vivo*. Closer examination of apoptosis revealed that TES had an apoptosis-inducing effect on CRC cells. Thus, these results suggest that TES plays a tumor suppressor role in CRC cells via inhibiting proliferation and promoting apoptosis in agreement with results from other tumors.

Human TES localizes to the cytoplasm as a component of focal adhesions and cell-cell contacts [13–18]. It contains a PET domain (found in a limited number of proteins, function has not been clarified to date [17]) and three tandemly arranged LIM domains (protein-interaction motifs that bind a wide range of different proteins [39]). TES interacts with a series of cytoskeletal proteins, such as Arp7A, alphaII-spectrin, talin, zyxin, Mena, EVL (Ena/vasodilator-stimulated, phosphoprotein-like protein), and actin [13–18]. Deletion of TES increases cell motility and decreases cell-cell connections, whereas TES overexpression causes the opposite effects [16, 18, 40]. To investigate whether TES has a similar effect in CRC cells, we evaluated the migration and invasion ability of TES-overexpressing and knockdown HCT116 and DLD-1 colon cancer cells. Increased TES expression reduced the migration and invasion ability of colon cancer cells *in vitro*. Additionally, we observed in the nine kinds of CRC cell lines studied (DLD-1, Caco-2, HT-29, SW48, SW480, SW620, HCT116, LoVo, and RKO) that cell types with higher TES expression (such as Caco-2 and HT-29) presented more circular colonies, while cell types with lower TES expression (such as SW480 and SW620) presented colonies more leaf-like at the edges under light microscopy.

To dissect the possible mechanisms by which TES suppresses proliferation and induces apoptosis of colon cancer cells, we identified the effect of TES on molecules involved in apoptosis. Overexpression of TES, decreased the levels of the anti-apoptotic proteins (Bcl-2 and survivin) and increased the levels of the pro-apoptotic proteins (p53, Puma, and Bax). These findings suggest that in CRC, the tumor suppressor effect of TES depends on apoptosis, or is at least partially associated with apoptosis.

We next investigated the MAPK and AKT signaling pathways – both known to be involved in CRC [41, 42]. We found that the phosphorylation of p38 was significantly increased by the overexpression of TES, but was attenuated by TES knockdown. The p38-MAPK pathway is a member of the conserved MAPK family of dual serine-threonine kinases, which respond to a variety of internal and external stimuli. The p38-MAPK pathway not only has important functions in inflammation and other cellular stresses, but also regulates cell proliferation and tumorigenesis. Still, since the p38 inhibitor did not reverse the migration and invasion suppressive effect of TES in CRC cells, the suppression effect of TES might not be dependent on the p38-MAPK signaling, but on the interaction with cytoskeletal proteins. The role of p38 in cancer remains controversial. Considerable data imply that p38 acts as a tumor suppressor, inducing cell-cycle arrest, differentiation, senescence, and apoptosis in various cancer types including prostate and colorectal cancer [43–53]. Mouse models of lung and liver cancers deficient in p38 exhibit higher tumor burden than their wild type counterparts [54–57]. Furthermore, p38 was found to be a marker for tumor dormancy and an inhibitor of self-renewal in several cancers, including melanoma as well as breast, prostate, and colorectal carcinomas [58]. Inhibition of p38 could enhance the chemo resistance of gastric cancer, which shares chemotherapy regimens with colorectal cancer [59]. Conversely, other reports have suggested that p38 is involved in cell survival and increased invasion in several advanced tumor types, including lung and prostate cancers [60–62], suggesting that increased p38-MAPK activity is correlated with poor prognosis. The inhibition of p38 was reported to reduce tumor growth in patient-derived xenografts from colon tumors [63].

Two opposite opinions have arisen. In a recent published study, it was reported that the response of CRC to p38 inhibitors is highly variable; while p38 inhibitors induces regression of one subgroup of CRCs, it stimulates growth of another subgroup. Additionally, it has been shown that PP2AC expression in the two different CRC subgroups determines the opposite effects of p38 inhibitors to the cells [64]. To some extent, these findings are consistent with the opinion that p38 signaling has a dual function in colorectal tumorigenesis [65]. Thus, the role and function of p38 in tumorigenesis is complex. It may function in a cell context-specific or cell type-specific manner, or may be affected by other unpredictable factors [46]. Even in the same kinds of cancers, p38 may act as either tumor suppressor or promotor (such as prostate cancer [52, 61], lung cancer [56, 62], and colorectal cancer [53, 63]). Our results suggest that TES overexpression could activate p38 MAPK, whereas pharmacological inhibition of p38 MAPK could alleviate the inhibitory effects of TES on CRC cells. Taken together, our findings suggest that the anti-proliferation and pro-apoptosis effects of TES are associated with the activation of the p38-MAPK signaling pathway, which is in good agreement with previous evidence for the tumor suppressor role of p38 in colorectal cancer. It is in opposition to the opinion that p38 acts as a tumor promotor in colorectal cancer simultaneously. The roles and mechanisms of p38 in colorectal cancer are still not very clear. Perhaps it plays different roles in sporadic CRC and colitis-associated CRC [65]. Alternatively, a single molecule could reverse its function in the same kind of cell [64].

In conclusion, this study first identified a tumor suppressor role of TES in CRC. Our findings indicate that overexpression of TES suppresses the development of CRC, whereas knockdown of TES promotes the progression of CRC. This effect appears to be related to the p38-MAPK signaling pathway – a finding that provides novel insights into the molecular mechanisms underlying CRC. Our results furthermore provide TES as a promising new therapeutic target for suppressing the onset of CRC.

## MATERIALS AND METHODS

All studies were conducted according to ethical standards, the Declaration of Helsinki, and national and international guidelines and have been approved by the authors' institutional review board.

### Patient samples, cell lines, and reagents

21 pairs of matched primary human colorectal carcinoma samples (the pathological diagnosis of these samples are all adenocarcinoma) and adjacent tumor-free, frozen tissue samples, which were obtained from patients at Wuhan Union Hospital who underwent surgical resection between 2013 and 2014, were used to test the mRNA expression and protein levels of TES. The clinical data of the 21 human subjects are summarized in Supplementary Table S1. Then, the correlation between histological grades or TNM stages and TES protein levels of the CRC samples were analyzed. Histological grading systems [66] and TNM stage groupings [67] for CRC were recommended by the “Protocol for the examination of specimens from patients with primary carcinoma of the colon and rectum [67]”. Samples were collected according to institutional protocols. All enrolled individuals were Han Chinese. This study was approved by the Ethics Committee of Union Hospital, Tongji Medical College, Huazhong University of Science and Technology (HUST). The data were analyzed anonymously.

Human FHC cells and CCD-18Co cells were cultured in a 1:1 mixture of Dulbecco's modified Eagle's medium (DMEM) and Ham's F12 medium (Invitrogen, Carlsbad, CA, USA) supplemented with 10% FBS (HyClone, Logan, UT, USA) and 1% penicillin-streptomycin (Gibco, Carlsbad, CA, USA) at 37°C in a 5% CO_2_ incubator (Thermo Fisher Scientific, Madison, WI, USA). Human DLD-1, Caco-2, HT-29, SW48, SW480, SW620, HCT116, LoVo, and RKO cells were cultured in McCoy's 5A medium (AppliChem, Darmstadt, Germany) supplemented with 10% FBS (HyClone, Logan, UT, USA) and 1% penicillin-streptomycin (Gibco, Carlsbad, CA, USA) at 37°C in a 5% CO_2_ incubator (Thermo Fisher Scientific Inc.). The correlation between Broder's grade or Duke's classification of the original tumors and TES protein levels of the CRC cell lines were analyzed. The differentiation of xenograft, Broder's grade, and Duke's classification of the original tumors of the CRC cell lines are listed in Supplementary Table S2. Cells were classified into two grades according to Broder's grades or differentiation of xenograft. The two-tiered grading system was recommended by the “Protocol for the examination of specimens from patients with primary carcinoma of the colon and rectum [66, 67]”.

HEK 293T cells were cultured in DMEM (high glucose; Invitrogen, Madison, WI, USA) supplemented with 10% FBS (HyClone, Logan, UT, USA) and penicillin-streptomycin (Gibco, Carlsbad, CA, USA) in a 5% CO_2_ incubator (Thermo Fisher Scientific Inc.) for packaging and expanding of lentiviral vectors.

SB203580 (p38 inhibitor) was purchased from Sigma-Aldrich (Saint Louis, Missouri, USA) and solubilized in DMSO according to the manufacturer's instruction. Lenti-TES cells (Lenti-TES-HCT116 and Lenti-TES-DLD-1) were treated with SB203580 at a final concentration of 50 μM, and then subjected to colony formation assays, cell proliferation assays, soft agar assays, cell migration and invasion assays, and cell apoptosis assays. Cells were allowed to form colonies for 14 days during which SB203580 was replenished every other day.

### Plasmid construction and RNA interference

The TES overexpression plasmid was generated by cloning the cDNA representing the complete open reading frame (ORF) of TES into the pSi-Flag vector (Invitrogen, Carlsbad, CA, USA). TES-F (CGCGGATCCATGGACCTGGAAAACAAAG) and TES-R (CCGCTCGAGCTAAGACATCCTCTTCTTAC) were chosen as primers. RNAi techniques were used to generate TES-knockdown clones. Oligos corresponding to the target sequences were annealed and cloned into the HpaI and XhoI sites of the pSicoR plasmid (Addgene, Cambridge, MA). TES-#1 (GAATGTCTCCATCAATACAG) and TES-#2 (GAACTCAATATTCCTGCTA) were chosen as target regions. All constructs were verified by sequencing.

### Virus generation and infection

Lentiviral vectors were transfected into HEK 293T cells in combination with the lentiviral packaging vectors pRSV-Rev, pMD2.G, and pCMV-VSV-G using Lipofectamine™2000 (Invitrogen, Carlsbad, CA, USA). After transfection for 48 h, supernatants were collected, filtered through a 0.4-μm filter, and directly used to transfect the HCT116 and DLD-1 cells to establish the TES-overexpression cell lines (Lenti-TES-HCT116 and Lenti-TES-DLD-1). The HCT116 and DLD-1 cells were transfected with empty vectors to generate the control cell lines (Lenti-NC-HCT116 and Lenti-NC-DLD-1). Clones were selected using 500 μg/mL of Geneticin (G418, Invitrogen, Carlsbad, CA, USA). Colonies resistant to G418 appeared within two weeks and single colonies were picked and expanded for another three weeks to obtain stable clone stock cells. The TES-knockdown cell lines pSicoR-shRNA-TES-#1 (shTES-#1-HCT116, and shTES-#1-DLD-1) and pSicoR-shRNA-TES-#2 (shTES-#2-HCT116, and shTES-#2-DLD-1) and the control cell lines (shRNA-HCT116, and shRNA-DLD-1) were established following the same procedures. Western blot analysis and real-time qPCR were performed to determine whether stable cell lines were successfully generated.

### RNA extraction and real-time qPCR

Total RNA was isolated using Trizol reagent (Invitrogen, Carlsbad, CA, USA) according to the manufacturer's instructions. 2 μg of total RNA was reverse transcribed using RNA PCR Kit (Takara Biotechnology, Otsu, Japan), and the resulting cDNA was used as a PCR template. The mRNA levels were determined by real-time qPCR with an ABI PRISM 7900 Sequence Detector system (Applied Biosystem, Foster City, CA, USA) according to the manufacturer's instructions and normalized to GAPDH gene expression. The experiment was performed in triplicate. The sequences of primers for real-time qPCR are listed in Supplementary Table S3.

### Western blot and antibodies

Total cells and tissues were lysed using RIPA buffer, and the protein concentration was determined with a BCA protein assay kit (Pierce Company, Rockford, IL, USA). Protein extracts were used for SDS-PAGE (Invitrogen, Carlsbad, CA, USA), and the proteins were transferred to a polyvinylidene fluoride membrane (Millipore, Billerica, MA, USA), which was incubated with various primary antibodies overnight at 4°C. After incubation with HRP-conjugated secondary antibodies (diluted 1:5000) for 1 h at room temperature, the membranes were treated with ECL reagents (170-5061, Bio-Rad, Hercules, CA, USA) prior to visualization using a FluorChem E imager (Cell Biosciences, San Jose, CA) according to the manufacturer's instructions. The specific protein expression levels were normalized to GAPDH on the same nitrocellulose membrane. The following primary antibodies and dilutions were used: anti-p53 (sc-126, diluted 1:500), anti-survivin (sc-17779, diluted 1:500) and anti-TES (sc-271184, diluted 1:500) were purchased from Santa Cruz Biotechnology (Santa Cruz, CA, USA); anti-ERK1/2 (#4695, diluted 1:1000), anti-phospho-ERK1/2^Thr202/Thr204^ (#4370, diluted 1:2000), anti-p38 (#9212, diluted 1:1000), anti-phospho-p38^Thr180/Thr182^ (#4511, diluted 1:1000), anti-JNK1/2 (#3708, diluted 1:1000), anti-phospho-JNK1/2^Thr183/Tyr185^ (#4668, diluted 1:1000), anti-AKT (#4691, diluted 1:1000), anti-phospho-AKT^Ser473^ (#4060, diluted 1:2000), anti-Bax (#2772, diluted 1:1000), anti-Bcl-2 (#2876, diluted 1:1000), anti-Puma (#4976, diluted 1:1000) were purchased from Cell Signaling Technology (Beverly, MA, USA); anti-GAPDH (MB001, diluted 1:1000) was purchased from Bioworld Technology (St Louis Park, MN, USA).

### Colony formation assay, soft agar assay and cell proliferation assays

For colony formation, as described in earlier studies [68], 1000 cells/well were seeded into 6-well cell culture plates and culture medium was replaced every three days. After 14 days of culture, cells were stained with crystal violet, and the number of visible colonies was counted.

For anchorage-independent growth assays in soft-agar, as described in earlier studies [69–71], Lenti-TES cells at 5 × 10^3^ cells/well were mixed with 0.05% Nobel agar (Fisher Scientific, Pittsburgh, PA) in growth medium which contains SB203580 at a final concentration of 50 μM and plated onto 6-well plates containing a solidified bottom layer (0.1% Noble agar in growth medium). After the incubation of cells for 14 days, the cells were stained with MTT [3-(4,5-dimethylthiazol-2-yl)-2,5-diphenyltetrazolium bromide], and the number of cell colonies was counted.

Cell viability and proliferation was determined by MTT assay and EdU cell proliferation assay. For the MTT assay, 1 × 10^3^ cells in 200 μL culture medium were plated into each well of 96-well plates. After culturing cells for an appropriate time, MTT was added into each well at a final concentration of 5 μg/mL and was allowed to remain in culture for 4 h before measurement. Optical density was measured at a wavelength of 570 nm and cell growth curves were determined according to the optical density value. For the EdU cell proliferation assay, as described previously [72], Cell-Light 5-ethynyl-2-deoxyuridine (EdU) DNA Cell Proliferation Kit (RIBOBio. Co. Ltd, Guangzhou, China) was used [73]. Briefly, cells (1×10^5^) were cultured in 24-well plates. After culturing for 24 h, the four kinds of transfected cells were exposed to 50 μM EdU for 2 h at 37°C. The cells were then fixed in 4% formaldehyde for 30 min at room temperature and permeabilized in 0.5% Triton X-100 for 10 min. Cells were washed with PBS, and each well was incubated with 200 μM Apollo reaction cocktail for 30 min, followed by Hoechst 33342 (200 mL per well) for staining nuclei. The stained cells were imaged under a fluorescence microscope (IX71, Olympus). The number of cells were counted using Image-Pro Plus v6.2 software.

### Cell migration and invasion assays

Migration of cells was evaluated by a wound-healing assay and uncoated Transwell^®^ inserts according to manufacturer instructions. For the wound-healing assay, as described in earlier studies [74–78], cells were plated and the monolayer of the cells was gently scratched with sterile micropipette tips, washed twice with PBS to remove cell debris, and incubated at 37°C with serum-free culture medium for serum starvation. The area of the gap at 0 h and the residual gap at 36 h after wounding of five random locations were acquired using a digital camera and microscope. A percentage of the cleared area at 36 h compared with 0 h after wounding was measured using Image-Pro Plus v6.2 software. For the uncoated Transwell^®^ assay, as described in earlier studies [59, 74, 79–81], the uncoated Transwell^®^ filter inserts with eight micron pores (BD Biosciences, Bedford, MA, USA) in 24-well cell culture plates were used. Cell invasion properties were measured by the Transwell^®^ Invasion assay (BD Biosciences, Bedford, MA, USA) along with chambers consisting of precoated Matrigel membrane Transwell^®^ filter inserts with eight micron pores in 24-well cell culture plates. 1 × 10^5^ cells were suspended and then seeded in the uncoated and precoated upper chambers of 24-well Transwell^®^ plates with FBS-free medium. Minimum essential medium containing 10% FBS in the lower chamber served as the attractant. After 24 h incubation at 37°C in a humidified 5% CO_2_ atmosphere, migrated and invasive cells were stained by 0.5% crystal violet solution for 15 min, observed by digital camera and microscope to take photomicrographs, and the cell migration was quantified by calculating the cell-covered area, as described in earlier studies [59], using Image-Pro Plus v6.2 software.

### Cell apoptosis assay

Cells were harvested 48 h after culturing in FBS-free medium, washed twice with PBS, and were resuspended in cold binding buffer. For apoptosis analysis, cells were stained with the Annexin V-fluorescein isothiocyanate (FITC)/7-amino-actinomycin D (7-AAD) staining system obtained from BD Biosciences (San Diego, CA, USA). For each assay, 1 × 10^5^ cells were incubated with 5 μL of Annexin V-FITC and 5 μL of 7-AAD in the dark for 15 min at room temperature. After adding 400 μL of binding buffer, the cells were analyzed within one hour by fluorescence-activated cell sorting using FACScan (BD Biosciences, San Diego, CA, USA). Cell populations were classified as viable (Annexin V negative, 7-AAD negative), apoptotic (Annexin V positive, 7-AAD negative or positive), or necrotic (Annexin V negative, 7-AAD positive). All samples were assayed in triplicate.

### Xenograft mouse model

All animal procedures were conducted in accordance with the Guidelines for the Care and Use of Laboratory Animals, which were prepared by the National Academy of Sciences, published by the National Institutes of Health, and approved by the Institutional Animal Care and Use Committee of HUST. Athymic nude mice (strain BALB/c nu/nu; Charles River Laboratories, Wilmington, MA, USA) were used for tumorigenesis studies. The mice were housed and maintained under specific pathogen-free conditions in facilities approved by the Association for Laboratory Animal of Hubei Province, China. 8 to 12 week old mice were used in accordance with institutional guidelines.

For the subcutaneous xenograft mouse model, HCT116 cells (1×10^6^ cells) were stably transfected with pSi-Flag-TES (Lenti-TES-HCT116), pSi-Flag-empty (Lenti-NC-HCT116), pSicoR-shTES-#1 (shTES-#1-HCT116), and pSicoR-shRNA (shRNA-HCT116) vectors and harvested from subconfluent cultures by a brief exposure to 0.25% trypsin and 0.02% EDTA. Trypsinization was stopped with RPMI 1640 medium containing 10% FBS, and the cells were washed once in serum-free RPMI 1640 medium and resuspended in Hanks Balanced Salt Solution. Only suspensions of single cells with > 90% viability were used for injection. These two pairs of corresponding HCT116 cells (Lenti-TES-HCT116 paired with Lenti-NC-HCT116 and shTES-HCT116-#1 paired with shRNA-HCT116) were subcutaneously injected at day zero into the right and left dorsal flank, respectively, of nude mice (6/group). Tumor diameter was measured every two days before harvest over the course of 36 days for the TES-overexpression pair group and 28 days for the TES-knockdown pair group. Tumor volume (mm^3^) was estimated by measuring the shortest (X) and longest (Y) diameters of the tumor using the formula: x^2^y/2.

### Statistical analysis

The statistical analysis was performed with the Statistical Package for Social Sciences (version 17.0; SPSS, Chicago, IL). All data are expressed as mean ± SD (standard deviation, SD). The differences were analyzed by one-way ANOVA for multiple comparison and Student's t-test for paired data between two groups. *P* < 0.01 was considered statistically significant.

## SUPPLEMENTARY FIGURES AND TABLES


