# Supplementary material for: TES inhibits colorectal cancer progression through activation of p38
Source: Oncotarget. 2016 Jun 13;7(29):45819–36. doi: 10.18632/oncotarget.9961 (PMC5216763; doi:10.18632/oncotarget.9961)
Supplement: Supplementary file 1 [file oncotarget-07-45819-s001.pdf]

TES inhibits colorectal cancer progression through activation of p38

SUPPLEMENTARY FIGURES AND TABLES

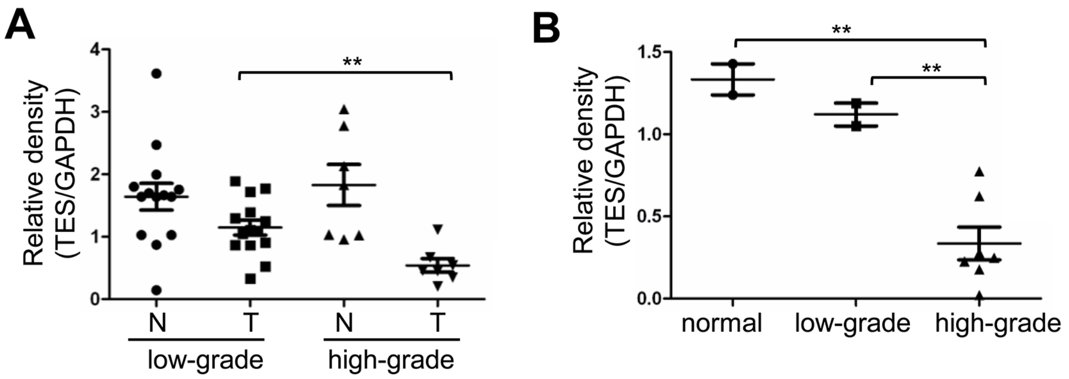

Supplementary Figure S1: Correlation between TES protein levels and histological grades of CRC samples and cell lines. A. Correlation between histological grades and TES protein levels of the CRC samples. N represents corresponding adjacent tumor-free tissue; T represents CRC tissue. Low-grade represents CRC patients with low histological grade. High-grade represents CRC patients with high histological grade. **B.** Correlation between Broder's grade of the original tumors and the TES protein levels of the CRC cell lines. Normal represents the cell group including FHC and CCD-18Co; Low-grade represents the cell group including HT-29 and Caco-2; High-grade represents the cell group including DLD-1, SW48, SW480, SW620, HCT116, LoVo, and RKO. **, $p < 0.01$. Data are plotted as the mean ± SD from five independent experiments. Bars indicate the standard deviation of the mean.

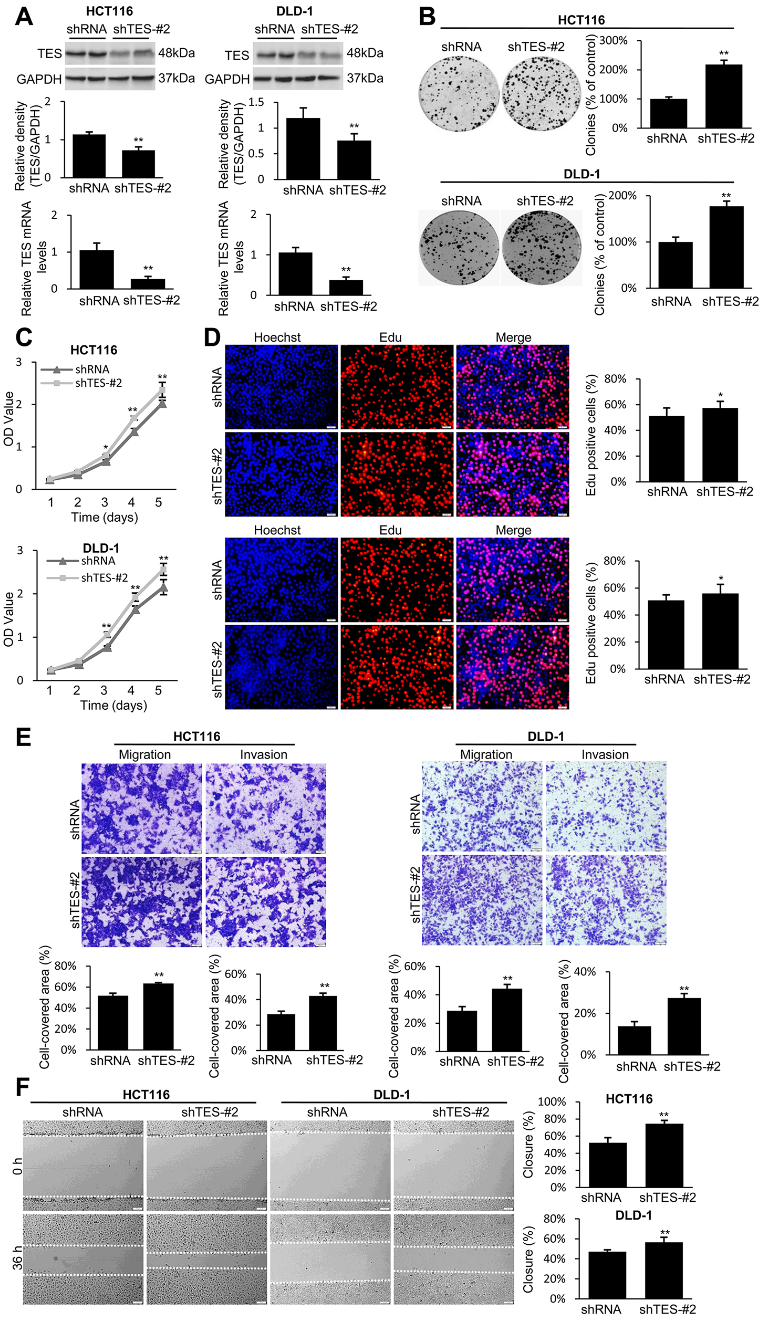

Supplementary Figure S2: The effect of shTES-#2 on proliferation, migration, and invasion in CRC cells. A. Confirmation of the protein levels and mRNA expression of TES in the shTES-#2 stably expressing HCT116 and DLD-1 cells by Western blot and real-time qPCR. GAPDH was used as endogenous control. **B.** Representative photographs of cell culture plates following staining with crystal violet for colony formation of the shTES-#2 stably expressing HCT116 and DLD-1 cells. Number of colonies was quantified and plotted as percent (%) relative to the control. **C.** The cell growth of the shTES-#2 stably expressing HCT116 and DLD-1 cells was determined by MTT assays at each time point. **D.** Representative profiles of EdU cell proliferation assay in the shTES-#2 stably expressing HCT116 and DLD-1 cells. Representative photographs were taken at 200× magnification. **E.** Cell migration and invasion was assessed after 24 h incubation by Transwell® assays. Representative photographs were taken at 100× magnification. **F.** Representative images of wound healing assay by scraping culture dishes using a pipet tip and closure after 36 h of culture. Representative photographs were taken at 100× magnification. * $p < 0.05$; ** $p < 0.01$. Data are plotted as the mean ± SD from five independent experiments. Bars indicate the standard deviation of the mean.

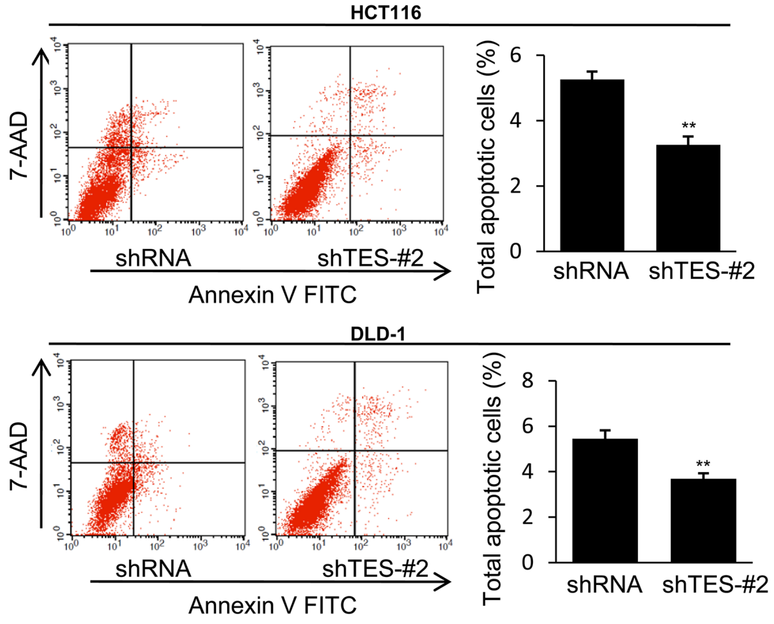

Supplementary Figure S3: The effect of shTES-#2 to apoptosis in CRC cells. The shTES-#2 stably expressing HCT116 and DLD-1 cells were grown over time respectively. Cell apoptosis was measured by flow cytometric analyses. Representative biparametric histogram showing cell population in apoptotic (top right and bottom right quadrants), viable (bottom left quadrant) and necrotic (top left quadrant) states. ** indicates significant differences, $p < 0.01$. Data are plotted as the mean ± SD from five independent experiments. Bars indicate the standard deviation of the mean.

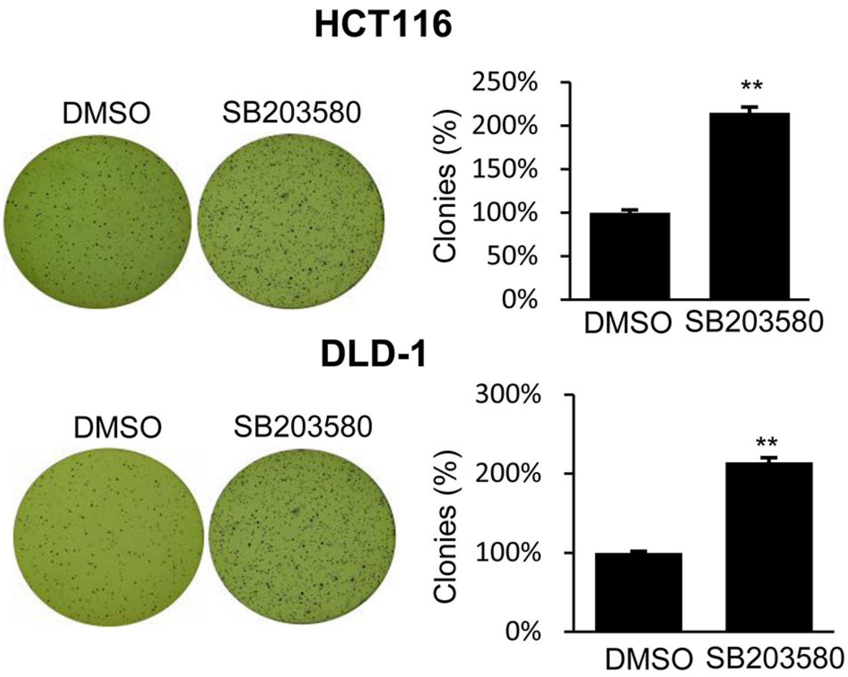

Supplementary Figure S4: Anchorage-independent growth assays in soft-agar. Representative photographs of cell culture plates following staining with MTT for colony formation of the TES-overexpressing HCT116 and DLD-1 cells in the presence or absence of p38 inhibitor. Number of colonies was quantified. ** $p < 0.01$. Data are plotted as the mean ± SD from five independent experiments. Bars indicate the standard deviation of the mean.

Supplementary Table S1: The major clinical data including histological grades and TNM stages of 21 human subjects and TES levels of the CRC tissue samples

No.	Gender	Age (Years)	T	N	M	histological grade	Tumor TES level (TES/GAPDH)
1	Male	62	2	1	0	Low-grade	1.89246
2	Male	48	4	1	0	High-grade	0.66822
3	Male	60	4	0	0	Low-grade	1.09384
4	Female	41	2	1	0	Low-grade	1.04884
5	Male	79	4	1	0	Low-grade	0.86372
6	Female	76	4	0	0	Low-grade	0.86809
7	Male	74	3	1	0	High-grade	0.54135
8	Male	56	2	1	0	Low-grade	1.25181
9	Female	40	4	0	0	High-grade	0.34694
10	Female	72	4	0	0	Low-grade	1.29930
11	Male	40	4	0	0	Low-grade	0.52622
12	Female	63	4	1	0	High-grade	0.20372
13	Female	53	1	0	0	High-grade	0.45950
14	Male	50	4	2	0	Low-grade	1.39457
15	Female	64	4	1	0	Low-grade	0.90680
16	Male	70	4	0	0	High-grade	1.11520
17	Female	51	2	0	0	High-grade	0.45318
18	Female	75	4	0	0	Low-grade	0.32808
19	Male	50	3	0	0	Low-grade	1.11109
20	Male	58	3	0	0	Low-grade	1.71962
21	Female	59	2	2	0	Low-grade	1.77426

Supplementary Table S2: The Broder's grade and Duke's classification of the original tumors and TES levels of the CRC cell lines

Cell line	Duke's stage	Broder's grade	Differentiation of xenograft	Histological grades
HCT116	ND	III/IV	Poor	High-grade
Caco-2	ND	II	Moderate	Low-grade
HT-29	ND	I	Moderately well	Low-grade
SW48	C	IV	Poor	High-grade
SW480	B	IV	Poor	High-grade
SW620	C	IV	Poor	High-grade
DLD-1	C	III/IV	Poor	High-grade
LoVo	C	IV	Poor/ Moderate	High-grade
RKO	ND	III/IV	Poor	High-grade

ND: not determined

Supplementary Table S3: The sequences of primers for real time qPCR

Gene	Primer Sequence (Forward and Reverse)
GAPDH	5′- CCACTCCTCCACCTTTGAC -3′
	5′- ACCCTGTTGCTGTAGCCA -3′
Bax	5'- TCTGACGGCAACTTCAACT -3'
	5'- TCCAATGTCCAGCCCAT -3'
Bcl-2	5'- GCCTTCTTTGAGTTCGGTG -3'
	5'- ACCTACCCAGCCTCCGTTAT -3'
survivin	5'- GCCCAGTGTTTCTTCTGCTT -3'
	5'- CCGGACGAATGCTTTTTATG -3'
p53	5'- TTTCCGTCTGGGCTTCT -3'
	5'- GCTGTGACTGCTTGTAGATGG -3'
PUMA	5'- GGAGACAAGAGGAGCAGCA -3'
	5'- CAGGGTGTCAGGAGGTGG -3'
TES	5'- GCCAAGAAGAATGTCTCCA -3'
	5'- TTGAAGGGTCCTGGTCAT -3'
